# Associations between self-reported healthcare disruption due to covid-19 and avoidable hospital admission: evidence from seven linked longitudinal studies for England

**DOI:** 10.1136/bmj-2023-075133

**Published:** 2023-07-19

**Authors:** Mark A Green, Martin McKee, Olivia KL Hamilton, Richard J Shaw, John Macleod, Andy Boyd, Srinivasa Vittal Katikireddi

**Affiliations:** 1 Geographic Data Science Lab, Department of Geography & Planning, University of Liverpool, Liverpool, UK; 2 MRC/CSO Social and Public Health Sciences Unit, University of Glasgow, Glasgow, UK; 3 Department of Health Services Research and Policy, London School of Hygiene and Tropical Medicine, London, UK; 4 Population Health Sciences, University of Bristol, Bristol, UK; 5 The National Institute for Health and Care Research Applied Research Collaboration West (NIHR ARC West) at University Hospitals Bristol and Weston NHS Foundation Trust, Bristol, UK

## Abstract

**Objectives:**

To examine whether there is an association between people who experienced disrupted access to healthcare during the covid-19 pandemic and risk of an avoidable hospital admission.

**Design:**

Observational analysis using evidence from seven linked longitudinal cohort studies for England.

**Setting:**

Studies linked to electronic health records from NHS Digital from 1 March 2020 to 25 August 2022. Data were accessed using the UK Longitudinal Linkage Collaboration trusted research environment.

**Participants:**

Individual level records for 29 276 people.

**Main outcome measures:**

Avoidable hospital admissions defined as emergency hospital admissions for ambulatory care sensitive and emergency urgent care sensitive conditions.

**Results:**

9742 participants (weighted percentage 35%, adjusted for sample structure of longitudinal cohorts) self-reported some form of disrupted access to healthcare during the covid-19 pandemic. People with disrupted access were at increased risk of any (odds ratio 1.80, 95% confidence interval 1.39 to 2.34), acute (2.01, 1.39 to 2.92), and chronic (1.80, 1.31 to 2.48) ambulatory care sensitive hospital admissions. For people who experienced disrupted access to appointments (eg, visiting their doctor or an outpatient department) and procedures (eg, surgery, cancer treatment), positive associations were found with measures of avoidable hospital admissions.

**Conclusions:**

Evidence from linked individual level data shows that people whose access to healthcare was disrupted were more likely to have a potentially preventable hospital admission. The findings highlight the need to increase healthcare investment to tackle the short and long term implications of the pandemic, and to protect treatments and procedures during future pandemics.

## Introduction

The covid-19 pandemic created unprecedented disruption to healthcare in the UK. Health facilities reoriented to care for surging numbers of patients with covid-19, initially through postponing or cancelling non-emergency treatment and diagnostic tests.^1^ People were deterred from seeking healthcare because of fear of being exposed to SARS-CoV-2 in health facilities,^2^ altruistic behaviours aimed at protecting the NHS,^3^ and reduced availability of face-to-face consultations.^4^ Collectively, these actions have resulted in fewer GP consultations,^5^ ^6^ diagnostic tests,^6^ ^7^ cancer referrals, diagnoses and treatments,^5^ ^8^ ^9^ cardiovascular treatment and surgery,^10^ elective and emergency hospital admissions,^11^ ^12^ ^13^ and increased waiting times for starting treatment.^14^ Additionally, more people have died at home during the pandemic,^15^ with changes in secondary care seeking behaviours potentially impacting these trends. Although these impacts are not unique to this country,^16^ ^17^ ^18^ ^19^ ^20^ the UK has fared much worse than many otherwise similar countries.

The extent of healthcare disruption has been described elsewhere,^21^ but our study links this disruption to empirically observed adverse health outcomes at the individual level. It is plausible that delays in diagnosis and treatment allow illnesses to progress to greater severity. The aim of our paper is to examine whether there is an association between people experiencing disrupted access to healthcare during the pandemic and risk of an avoidable hospital admission. Avoidable hospital admissions are unplanned admissions that could potentially have been prevented through timely care delivered in the community. The concept is used as a warning sign for failings in health system performance and is a key metric used in the NHS.^22^ ^23^ ^24^ ^25^ We hypothesise that people whose care was disrupted during the pandemic are more likely to have an avoidable hospital admission. Given that pandemic disruption has affected the lives of everyone, this approach allows us to evaluate the overall impact of disruption rather than focusing on discrete services whose study might obscure the overall effect of society wide disruption.

Capturing individual experiences of healthcare disruption from electronic health records is difficult, but they can be identified in longitudinal surveys. We linked data on participants in longitudinal cohorts with their electronic health records to examine the impact of any disruption they experienced due to covid-19 on health outcomes at an individual level.

## Methods

### Data

We used cohort data from seven UK population based longitudinal studies linked to electronic health records from NHS Digital for England. These studies included five birth cohorts (1946 National Survey of Health and Development, 1958 National Child Development Study, 1970 British Cohort Study, Next Steps, and Millennium Cohort Study) and two age heterogenous studies (English Longitudinal Study of Ageing and Understanding Society). Appendix table S describes each cohort. The cohort data were accessed using the UK Longitudinal Linkage Collaboration (UK LLC). The UK LLC trusted researcher environment hosts deidentified data from many longitudinal population studies and systematically links these to participants’ health, administrative, and environmental records within a secure analysis environment. Ethical approval for the project was granted by the University of Liverpool’s Research Ethics Board (reference 10634).

Each cohort study sent surveys to members of their cohorts inquiring about their experiences during the covid-19 pandemic, supplementing their prepandemic data collection processes. Data from all participants who responded to these surveys across the cohorts were pooled, giving a total sample size of 41 439 people. Combining cohorts brings value through improving the representativeness of the data and increasing statistical power.^21^ We excluded people living outside England because data linkage was not possible (n=5975). We further excluded people who did not consent to linkage or for whom linkage was not possible (n=5911). We excluded all those who died during the study period (n=277). The total analytical sample size was n=29 276 (appendix table A breaks down sample sizes by cohort).

### Outcomes

Linkage of cohort members to electronic health records was conducted by the UK LLC. Electronic health records from NHS Digital included civil registration of deaths, secondary care (hospital episode statistics admitted patient care), and vaccination status. We selected records between 1 March 2020 (which we define as the start of NHS disruption) and 25 August 2022 (end of available data).

We selected two measures of unplanned avoidable hospital admissions commonly used for evaluating NHS performance: ambulatory care sensitive, and emergency urgent care sensitive conditions.^22^ ^23^ Ambulatory care sensitive conditions are those that can be, in theory, treated through community care and therefore should not require hospital admission.^23^ ^26^ We used an overall measure for any ambulatory care sensitive condition and also stratified them by type into acute (eg, cellulitis, dental caries, rickets, gastric ulcer), chronic (eg, hypertension, angina, asthma), and vaccine preventable (eg, mumps, measles, influenza). Emergency urgent care sensitive conditions are acute exacerbations of urgent conditions that will potentially result in hospital admission, but that the NHS should be trying to treat within the community to minimise the need for hospital care.^23^ ^26^ Code lists were designed to match NHS Digital’s approach (openly available at https://www.opencodelists.org/users/mgreen/) and we selected any emergency hospital admission during the study period where codes were present in the primary diagnosis field. Covid-19 was excluded from this definition of avoidable hospital admissions. We also included an outcome of whether a person had any hospital admission during the study period to contextualise our findings. This approach gave us a total of six outcome variables that we considered in the main analyses. All outcome variables in the main analysis were binary outcomes.

We used covid-19 vaccination status (binary—person had or had not received two covid-19 doses by the end of the study period) as an outcome variable in a falsification test.^27^ ^28^ We did not expect an association between experiences of healthcare disruption and vaccination uptake because vaccine delivery was prioritised and therefore less disrupted. This method provides an imperfect, but valuable, instrumental indicator to assess the role of unobserved confounding in our models. We hypothesised that if there were any residual confounding, we would find an association here because it would confound self-reported disruption to healthcare and falsification variables in similar ways.

### Self-reported disruption to healthcare

Our primary variable was whether people self-reported any disruption to healthcare (cancelled or postponed care, changes to planned or existing treatments), which was measured across all waves of data collection. This variable was based on questions asked in each longitudinal population study about participants’ experiences of healthcare during the pandemic. Disruption was defined where the question explicitly asked about experiences of disruption in accessing health services, or where people had appointments, treatment, or surgery booked and they reported that these were changed, postponed, or cancelled. We further stratified type of disruption into appointments (eg, visiting their GP or an outpatient department), procedures (eg, surgery, cancer treatment), and drugs. This approach allowed us to examine the different pathways through which disruption affected people. Appendix table B gives descriptions of questions asked in surveys and how they were harmonised.

### Control variables

We made adjustments for the longitudinal cohorts (categorical variables). Variables were selected that were consistent and comparable across longitudinal population studies, limiting the measures we could include (see appendix table A for full description of this harmonisation process). We chose the most recent value for each measure during the covid-19 waves (although selecting older responses did not change the main findings). Personal characteristics of age, sex (male or female), and ethnicity (white or ethnic minority) were included to account for demographic differences. Age was included as a linear and quadratic term to account for the nonlinear increase in risk of hospital admission with age. Racially minoritised ethnic groups often face structural barriers in accessing healthcare, with avoidable hospital admissions being higher for some ethnic groups.^24^ ^29^ The inconsistent ethnic categorisations used meant that we had to combine them into a simplistic binary definition. Socioeconomic position was measured using housing tenure (defined as owned house outright or with mortgage, renting (social or private), or other tenure) and neighbourhood socioeconomic deprivation (2019 index of multiple deprivation provided through linkage by NHS Digital). Including multiple measures of socioeconomic position is important to capture different dimensions of social stratification. Tenure accounts for accumulated wealth and neighbourhood deprivation measures for the broader socioeconomic context of a person’s life. Both measures have been shown to be independently associated with health outcomes, including avoidable hospital admissions.^23^ ^30^ ^31^

Adequately measuring health and comorbidity was pertinent because sicker people are likely to be at greater risk of hospital admissions. Adjusting for health status is complex and we account for several aspects. Comorbidity was measured using the Charlson comorbidity index with Quan weights calculated from hospital episode statistics one year before the pandemic (1 March 2019 to 29 February 2020).^32^ This index describes the extent of comorbidity of people across 16 health conditions and is predictive of mortality,^32^ as well as ambulatory care sensitive conditions.^33^ ^34^ Self-rated health status (excellent or good, fair or poor) was included as an indicator of physical health. This measure is associated with objective health outcomes, including mortality^35^ ^36^ ^37^ and avoidable hospital admissions.^38^ ^39^ While health status might sit on our causal pathway between disruption and health outcomes (eg, people with poor health are more likely to have an avoidable hospital admission and could have greater healthcare needs, making them more affected by disrupted access to healthcare), we treat it here as a key confounder that needs to be adjusted for.

We also assessed alternative health and comorbidity outcomes as sensitivity analyses. The Elixhauser index was used as an alternative to the Charlson comorbidity index to avoid relying on a single composite index.^40^ We included presence or absence of four self-reported health conditions (asthma, cancer, diabetes, and hypertension) as covariates to measure comorbidity. Because records were collected during the covid-19 surveys, we cannot be sure that they were present before experiences of disruption, therefore we include these as sensitivity analyses because they might act as mediators.

### Statistical analyses

Descriptive statistics were calculated to provide summary measures of our data. In the main analyses, we used logistic regression models because our six outcome variables were all binary outcomes. For each outcome, we ran two logistic regression models: an unadjusted model for which measures of self-reported disruption to healthcare were the only independent variables, and a fully adjusted model that included all measures and control variables together. Model coefficients were converted to odds ratios to help interpret associations and we present these data visually.

In the main analysis, we considered any outcome during the study period because we were unsure when experiences of disruption occurred (people were only asked to report if they had experienced disruption at any point). Three sensitivity analyses were undertaken to assess the robustness of the modelling framework. Firstly, we limited our six outcome variables to hospital admissions that only took place after the final survey date. With this approach, we could be certain that avoidable hospital admissions happened after experiences of disrupted access to healthcare. We used the same logistic regression model specification as the main analyses for our six outcomes. Secondly, we used a Cox regression model as an alternative method for modelling each of the six outcomes. Rather than using a binary measure of whether a person had an avoidable hospital admission, we used a time to outcome measured from the last survey date; this was because follow-up times were not consistent across cohorts because of differences in the last survey date. Thirdly, we used a logistic regression model to predict whether a person was vaccinated or not (binary outcome). This falsification test assessed potential residual confounding in our model because many shared confounders are likely to apply to this outcome (eg, people who are more likely to seek healthcare are more likely to be vaccinated and to be admitted to hospital).

All analyses accounted for the sampling design of each survey, including sample weights that account for representativeness, attrition, and non-response (sample weights, primary sampling units, stratums, and finite population correction factor were adjusted for). Appendix table C presents the numbers of missing values across our variables. Missing values for each variable were imputed using polytomous regression with all other measures and control variables. We also report analyses using only complete cases as a sensitivity analysis. Model assumptions for both types of regression models were checked and we did not identify any problems. All analyses were conducted using R statistical software and the code is openly available (https://github.com/markagreen/healthcare_disruption_LLC).

### Patient and public involvement

No specific engagement for the project. Feedback on the remit of the project and a lay abstract were provided from patient and public involvement groups as part of the UK LLC data access application review process. This helped to refine our research question, which was previously unclear, as well as how to communicate the problem we were addressing.

## Results

### Main analyses

Table 1 presents summary statistics of our analytical sample. Each outcome was uncommon during our study period. By 25 August 2022, 14% (weighted percentage) of participants had a hospital admission; 3% of participants were admitted for an ambulatory care sensitive condition. Among these admissions, vaccine preventable admissions were the least common (0.8%). Thirty-five per cent of participants reported experiencing any form of disruption in their access to healthcare due to covid-19. Disruption was most commonly experienced in accessing appointments (26%), followed by procedures (18%). Few people experienced disruption in their access to drugs (6%). Table 2 presents summary statistics for control variables. Participants who experienced some form of disrupted access to healthcare were older, had poorer health, and were more likely to live in the most deprived areas. Participants with linked data experienced more disruption than those with unlinked data, although differences were only small (1-2% more) and therefore are unlikely to strongly bias our observations (appendix table D). Missing data were higher for disrupted access to drugs (30.5%) and procedures (18.8%; appendix table C).

**Table 1:** Summary statistics for outcome variables and measures in the pooled sample

Measure	Frequency	Unweighted percentage	Weighted percentage*
Total admissions	3618	12.36	13.65
Ambulatory care sensitive any	780	2.66	3.36
Ambulatory care sensitive acute	347	1.19	1.32
Ambulatory care sensitive chronic	369	1.26	1.39
Ambulatory care sensitive vaccine preventable	94	0.32	0.78
Emergency urgent care sensitive	625	2.13	2.37
Two covid-19 vaccine doses	27 513	93.98	92.64
Disruption—any	9742	33.28	34.79
Disruption to appointments	7456	25.47	26.20
Disruption to drugs	1568	5.36	5.86
Disruption to procedures	5292	18.08	18.12

* Values adjusted for sample structure of each longitudinal study.

**Table 2:** Summary statistics for control variables for participants overall and for those experiencing disruption or no disruption to healthcare

Measure	Total				Any disruption				No disruption		
	Frequency	Unweighted value	Weighted value*		Frequency	Unweighted value	Weighted value*		Frequency	Unweighted value	Weighted value*
**Main analyses**											
Age, mean (SD)	—	52.9 (18.6)	52.9 (19.5)		—	57.9 (17.5)	59.6 (17.6)		—	50.4 (18.6)	49.4 (19.6)
Female	15 721	53.7	48.5		5334	54.8	50.7		10 387	53.2	47.3
Male	13 555	46.3	51.5		4253	43.7	49.3		9302	47.6	52.7
White	26 760	91.4	92.4		8835	90.7	93.1		17 925	91.8	92.0
Not white	2516	8.6	7.6		752	7.7	6.9		1764	9.0	8.0
Do not own home	14 565	50.1	49.5		4648	47.7	47.1		9917	50.8	50.7
Own home	14 711	49.9	50.5		4939	50.7	52.9		9772	50.0	49.3
Poor health	6202	78.8	24.3		3354	34.4	38.7		2848	14.6	16.9
Good health	23 074	21.2	75.7		6233	64.0	61.3		16 841	86.2	83.1
IMD rank											
1 (most deprived)	3638	12.4	16.4		1237	12.7	18.8		2401	12.3	15.2
2	4784	16.3	17.8		1651	16.9	18.5		3133	16.0	17.5
3	6038	20.7	20.1		1988	20.4	20.1		4050	20.7	20.0
4	7144	24.4	22.4		2331	23.9	21.3		4813	24.6	22.9
5 (least deprived)	7672	26.2	23.4		2380	24.4	21.3		5292	27.1	24.4
Charlson comorbidity index, mean (SD)	—	0.1 (0.5)	0.1 (0.5)		—	0.2 (0.7)	0.2 (0.7)		—	0.1 (0.4)	0.1 (0.4)
**Sensitivity analyses**											
Elixhauser index, mean (SD)	—	0.2 (0.7)	0.2 (0.8)		—	0.4 (1.0)	0.5 (1.0)		—	0.1 (0.5)	0.1 (0.5)
Asthma	3044	10.4	10.7		1193	12.2	13.1		1851	9.5	9.5
Cancer	833	2.8	2.6		482	4.9	4.7		351	1.8	1.5
Diabetes	1637	5.6	6.7		898	9.2	10.9		739	3.8	4.6
Hypertension	4398	15.0	17.2		1941	19.9	25.1		2457	12.6	13.1

Data are numbers and percentages (categorical or binary measures) unless indicated otherwise.

IMD=2019 index of multiple deprivation; SD=standard deviation.

* Values adjusted for sample structure of each longitudinal study.

**Fig 1:**
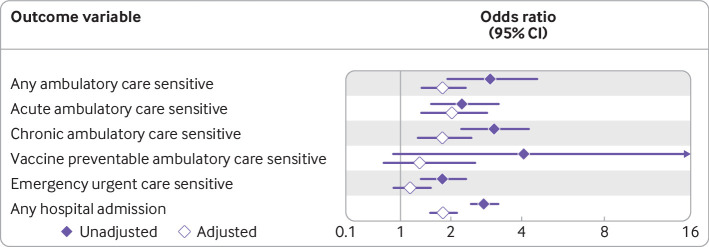
Model summary statistics for logistic (binomial) regression exploring associations between experiences of healthcare disruption and whether people had avoidable hospital admissions. Models adjusted for age, age squared, sex, ethnicity, housing tenure, self-rated health status, Charlson comorbidity index, and longitudinal cohort

Figure 1 presents results from a series of logistic regression models relating experiences of healthcare disruption to each of our six outcome variables (see appendix table E for full model output). We found positive associations between experience of healthcare disruption and five of our outcomes in the unadjusted models, with no clear association with vaccine preventable ambulatory care sensitive hospital admissions. After adjusting for known explanatory factors, positive associations remained for four outcomes and were attenuated for emergency urgent care sensitive hospital admissions. People who experienced any form of healthcare disruption had 80% higher odds of being admitted to hospital for any ambulatory care sensitive condition (odds ratio 1.80, 95% confidence interval 1.39 to 2.34), twofold higher odds of being admitted for an acute ambulatory care sensitive condition (2.01, 1.39 to 2.92), and 80% higher odds of being admitted for a chronic ambulatory care sensitive condition (1.80, 1.31 to 2.48). We also found that people who experienced disrupted access to healthcare had 82% higher odds of being admitted to hospital during the study period (1.82, 1.55 to 2.14).

**Fig 2:**
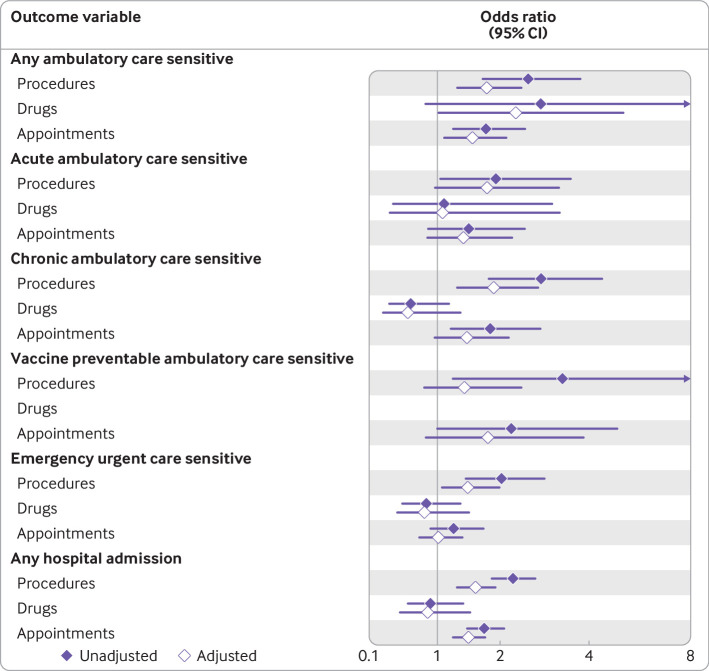
Model summary statistics for logistic (binomial) regression exploring associations between experiences of three types of healthcare disruption (procedures, drugs, and appointments) and whether people had avoidable hospital admissions (by type). Models adjusted for age, age squared, sex, ethnicity, housing tenure, whether person had covid-19, self-rated health status, Charlson comorbidity index, and longitudinal cohort. Results for vaccine preventable ambulatory care sensitive conditions and disruption to drugs were not robust because of a few issues

We then investigated how the type of healthcare disruption experienced was associated with our six outcome variables (fig 2; see appendix table F for full model output). People who experienced disrupted access to procedures had 77% higher odds of being admitted to hospital for any ambulatory care sensitive condition (odds ratio 1.77, 95% confidence interval 1.30 to 2.41), 88% higher odds of being admitted to hospital for a chronic ambulatory care sensitive condition (1.88, 1.28 to 2.75), 45% higher odds of an emergency urgent care sensitive admission (1.45, 1.05 to 1.99), and 57% higher odds of any hospital admission (1.57, 1.28 to 1.92). People who experienced disruption in accessing appointments had 52% higher odds of hospital admission for any ambulatory care sensitive condition (1.52, 1.09 to 2.12), and 46% higher odds of any hospital admission (1.46, 1.21 to 1.75). Finally, people who experienced disrupted access to drugs had more than twofold higher odds of being admitted to hospital for any ambulatory care sensitive condition (2.29, 1.02 to 5.10), although the confidence intervals were wide suggesting some caution in interpreting this result. We did not find any clear associations between this type of disruption and any other outcome.

### Sensitivity analyses

We excluded all hospital admissions that occurred before the last survey date to ensure that any self-reported disruption occurred before each outcome. Appendix table G presents summary statistics for these outcome variables. Irrespective of the statistical model used, the analyses were in agreement with the analyses presented in the main analyses (appendix tables H-K). The main difference was that the Cox regression model detected a greater number of associations between self-reported disruption to healthcare and outcomes, which were always positive associations.

We assessed the impact of controlling for additional health measures on our findings. Replacing the Charlson comorbidity index with the Elixhauser index did not materially change any associations (appendix tables L and M). When we also controlled for asthma, cancer, diabetes, and hypertension, the findings were in agreement with the main analyses (appendix tables N and O). These findings suggest that our analyses were robust to alternative specifications of controlling for health.

We used a falsification test to investigate whether our measures of healthcare disruption were associated with covid-19 vaccination uptake (appendix table P). Looking at overall experiences of healthcare disruption we found a positive association in unadjusted analyses, which was attenuated after adjustment. When considering the type of healthcare disruption, we again detected associations in unadjusted analyses that disappeared after adjustment. This finding suggests a low risk of residual confounding in our fully adjusted associations.

Finally, we reran our main analyses for complete cases only. When considering any experience of disrupted access to healthcare, associations with any and acute ambulatory care sensitive admissions, and any hospital admission were consistent (appendix table Q). There was a reduction in the point estimate for chronic ambulatory care sensitive admissions (odd ratio 1.48, 95% confidence interval 0.93 to 2.36). The model detected an association with vaccine preventable ambulatory care sensitive admissions (2.44, 1.15 to 5.21), although the confidence intervals were wide. We next examined differences in analysing type of healthcare disruption (appendix table R). Point estimates were in relative agreement with the main analyses. The model also detected a positive association between disruption to procedures and acute ambulatory care sensitive admissions (1.58, 1.03 to 2.44).

## Discussion

### Principal findings

Our study used linked individual level data to examine the impact of healthcare disruption on avoidable hospital admissions. We estimate that 35% of people in England experienced disrupted access to healthcare, with disruption to appointments (eg, visiting a GP or an outpatient department) being most common. Overall, people who reported any form of disruption in accessing healthcare were more likely to have been admitted to hospital for an avoidable or potentially preventable condition between 1 March 2020 and 25 August 2022. We found an increased risk in hospital admission for any (80% higher odds), acute (twofold), and chronic (80%) ambulatory care sensitive conditions. People who reported disruption in accessing drugs or appointments were more likely to be admitted to hospital for any ambulatory care sensitive conditions. People who reported disruption in accessing procedures were more likely to be admitted to hospital for any, chronic ambulatory care sensitive, or emergency urgent care sensitive conditions.

### Interpretation

There are several potential explanations for why disrupted access to healthcare was associated with avoidable hospital admissions. Appointments with healthcare professionals provide people with opportunities to seek advice, access secondary care, have diagnostic tests, and receive treatment.^5^ ^6^ ^19^ Disruptions could delay care that is needed, with people needing hospital admission as diseases progress (eg, presenting at later disease stages that are harder to treat). In particular, sudden changes in health often prompt people to seek a consultation,^41^ which might explain why we found an association with any ambulatory care sensitive conditions. Similarly, disruptions to procedures (eg, surgery, treatment) might lead to exacerbations of existing and longer term conditions, or disease progression that would otherwise have been treated,^16^ ^20^ ^23^ which could be why we found associations with chronic ambulatory care sensitive conditions. We are cautious about drawing too many conclusions from our data about what should be done to prevent disruptions. However, our finding on disrupted access to procedures being associated with avoidable hospital admissions is intuitive. Treatment and surgeries are less easy to deliver safely during a pandemic, whereas consultations and access to drugs can be provided remotely, especially given the opportunities of online working. Future research should tease out these specific pathways where disruption leads to an avoidable hospital admission to identify mechanisms that could mitigate the effects of service disruption.

Few people experienced problems with obtaining drugs (6%), which were also rarely associated with avoidable hospital admissions. Our findings suggest that drug supply was resilient during the pandemic. Even during periods of greatest disruption, when there were lockdowns, pharmacies were deemed essential services and remained open in the UK. While pharmacies did experience some issues with staff sickness and drug shortages,^42^ they adapted successfully, helped by remote GP consultations and home deliveries.^4^ Other evidence suggests that while repeat prescriptions were not affected by the pandemic, the rate of new prescriptions in England fell by 30%.^6^ The disruption in accessing GP appointments possibly led to reduced opportunities for adjustments to drug treatments, therefore reducing treatment effectiveness. This issue could have influenced the risk of avoidable hospital admissions.

Our findings show the need to increase investment in the health system to counter the negative effects of healthcare disruption resulting from the covid-19 pandemic. While NHS activity has returned to some extent, it has not returned to 2019 levels^29^ and the NHS has struggled to clear the backlog of treatments, diagnostic tests, procedures, and appointments.^43^ More recent disruption during winter 2022-23, with low rates of staff retention, chronic underfunding, healthcare worker strikes, high levels of staff illness, high prevalence of influenza and covid-19, and persisting waiting lists have compounded the pandemic related disruption.^43^ ^44^ The challenging economic context of high inflation and Brexit have been barriers to increased funding. This situation has placed the NHS in a difficult position as it seeks to tackle its legacy of underinvestment in labour and capital, both of which are critical in responding to the longer term impacts of healthcare disruption caused by the pandemic.^5^

### Strengths and limitations of this study

Our study has several strengths. We combined data from seven individual level longitudinal studies, which are drawn from independent nationally representative samples. Using only a single study would have restricted our sample size for events or limited analyses to certain demographic groups. The ability to systematically link self-reported data on disruption experienced to participants’ electronic health records within the UK LLC has been crucial to overcome previous barriers of linking survey data with health records.^45^ By combining individual level longitudinal studies and electronic health records we complement their individual strengths (personal details about people alongside objective hospital admissions data). Importantly, our results contrast with research using electronic health records alone, which showed falls in avoidable hospital admissions during periods of greatest disruption.^29^ These different insights show how analysis of population level routine records can be misleading when they do not have the same level of detail as linked individual level survey data.

Our analyses are observational and have limited ability to draw causal inferences. While our questions about healthcare disruption were specifically asked as part of surveys about experiences of the pandemic, we did not have any data on people’s difficulties in accessing healthcare before the pandemic. Prepandemic disruption to accessing healthcare across the NHS was minimal compared with the unprecedented nature and extent of disruption the pandemic brought in accessing healthcare, suggesting that our questions on experiences of this disruption are valid. We were unable to link specific experiences of disruption to particular adverse events, and not all avoidable hospital admissions would have been caused by disruption of care. The measured outcomes might not have occurred after self-reported healthcare disruption in our main analysis; however, sensitivity analyses were restricted to only outcomes after self-reported healthcare disruption, which suggests little difference in the findings. Because healthcare disruption was self-reported, it might be subject to bias. For example, ‘plaintive set’ can influence self-reported measures and could have inflated reports of disruption. When this reporting bias also influences outcome measures in the same direction, biased associations can arise. Hospital admission depends on a person presenting to a health facility and complaining of their condition. We have shown elsewhere that this situation can lead to bias,^46^ although it might have been less of a risk during our study given the general reluctance of hospitals to admit patients during the study period.^29^

Inconsistency of measures across cohorts limited our ability to control for potential confounders. Exclusion through non-participation in surveys might have introduced bias in our sample. Pooling independently representative birth cohorts and age heterogenous longitudinal population studies together might not generate a representative pooled sample (eg, birth cohorts might introduce over representation of some ages). Our pooled sample under represented people from ethnic minority backgrounds and more deprived areas, which might be partly explained by four of the seven longitudinal population studies targeting middle aged or older adults. However, we applied sample weights, and even non-representative studies tend not to show differences in their estimates of associations.^47^ Some bias could have been introduced by data linkage owing to incomplete or incorrect matching,^45^ ^48^ which could disproportionately impact on marginalised groups and people who might have migrated to other parts of the UK. Biases might have been introduced when study participants did not consent to linkage to their health records,^48^ although the impacts on our measures was limited (appendix table D). Missing data were also moderate for some measures (eg, disrupted access to drugs was 30.5%), which might have introduced bias, although imputed and complete case analyses produced similar findings. Because our outcome variables were uncommon, we were unable to explore whether experiences of healthcare disruption were greater in particular population subgroups (eg, by ethnicity or deprivation). This issue is pertinent because the topics we examine are not evenly felt across the UK. For example, healthcare disruption was disproportionately experienced in socioeconomically deprived communities,^5^ ^11^ ^21^ with avoidable hospital admissions also higher in deprived areas.^49^ The rarity of our outcomes produced wide confidence intervals; therefore, even with a large pooled sample, power is limited. Exploring how disrupted access to healthcare mediated the association between social inequalities and health is an important research gap.

Unplanned hospital admissions might only occur after a long period, stretching beyond our study period,^21^ and so we could have underestimated the impacts of healthcare disruption. Therefore, following the experiences of cohorts over longer time periods would be useful to determine whether this is the case. Additionally, the sensitivity of avoidable hospital admissions to health system performance is questionable because they might be affected by issues beyond the control of health systems (eg, socioeconomic deprivation).^23^ ^50^ Future research should investigate other outcomes, including moving away from composite indicators, to understand the pathways through which disruption impacts people. The current expansion of the UK LLC to include additional longitudinal studies and a larger participant sample size might enable such research.

The use of vaccination status as a falsification test is a strength, but cannot definitively confirm a lack of residual confounding.^27^ ^28^ People who were not vaccinated might be less connected to the health system and therefore less likely to experience disruption. Future research might consider more robust indicators.

### Conclusions

The external shock to the health system caused by the covid-19 pandemic seriously disrupted access to healthcare and this impact is having negative impacts on hospital admissions that could potentially be preventable. With developing narratives on how to respond to a pandemic, continued disruption to the NHS, and how to ‘build back better’, our study highlights the need for increasing healthcare investment to tackle the short and long term implications of the pandemic.

**What is already known on this topic**

Extensive research has described the extent of disruption in accessing healthcare services and treatment during the covid-19 pandemic
Studies are needed evaluating whether experiences of disruption are associated with negative health outcomes

**What this study adds**

People who experienced disrupted access to healthcare (including appointments and procedures) during the covid-19 pandemic were more likely to have potentially preventable hospital admissions
Reducing the backlog from covid-19 disruption is vital to tackle the short and long term implications of the pandemic

## Data Availability

Datasets used in the study contain sensitive and personal data, which means that data cannot be openly shared. All data can be accessed by accredited researchers through application to UK Longitudinal Linkage Collaboration (https://ukllc.ac.uk/). R scripts to replicate the analyses presented in the paper can be openly accessed at https://github.com/markagreen/healthcare_disruption_LLC.

## Web extra

Extra material supplied by authors

Web appendix: Appendix

## Notes

Contributors: MAG and SVK developed the idea of the study from conception. MM and JM helped to refine the research questions and study design further. MAG, with support from OKLH, RJS, and AB, accessed and manipulated the data into an analysis ready format. MAG, with support from OH, RJS, and SVK, conducted the analyses. MAG, MM, and SVK wrote the initial draft of the manuscript, with OKLH, RJS, and JM helping to refine further. All authors approved the paper. MAG acts as guarantor for the paper. The corresponding author attests that all listed authors meet authorship criteria and that no others meeting the criteria have been omitted.

The lead author (the manuscript’s guarantor) affirms that the manuscript is an honest, accurate, and transparent account of the study being reported; that no important aspects of the study have been omitted; and that any discrepancies from the study as originally planned (and, if relevant, registered) have been explained.

Dissemination to participants and related patient and public communities: Plain language summaries of the results will be shared on both the UK Longitudinal Linkage Collaboration and National Core Studies Longitudinal Health and Wellbeing websites. Patient and public involvement groups run in each of these projects will input on the design and content of these summaries. A policy brief has been written and will be openly shared on the National Core Studies Longitudinal Health and Wellbeing website (as well as e-mailed to relevant policy contacts). The paper will be shared on social media via Twitter on the personal accounts of the authors. An infographic has also been created to share via social media. We will release a press release of the main findings to share the results more widely.

Provenance and peer review: Not commissioned; externally peer reviewed.
