# Supplementary material for: Associations between self-reported healthcare disruption due to covid-19 and avoidable hospital admission: evidence from seven linked longitudinal studies for England
Source: BMJ. 2023 Jul 19;382:e075133. doi: 10.1136/bmj-2023-075133 (PMC10354595; doi:10.1136/bmj-2023-075133)
Supplement: Supplementary file 1 — Web appendix: Appendix [file grem075133.ww.pdf]

Appendix

Table A: Analytical sample size by longitudinal population study.

Cohort	Sample size	Percentage
1970 Birth Cohort	4122	14.1
English Longitudinal Study of Ageing	5567	19.0
Millennium Cohort Study	3350	11.4
1958 Birth Cohort	4725	16.1
NextSteps	2891	9.9
1946 Birth Cohort	1813	6.2
Understanding Society	6808	23.3
Total	29276	100

Note on pooling longitudinal population studies: The UK LLC provides the novel platform to systematically link study-collected data with participants' routine health records and to integrate diverse longitudinal data into a single pooled dataset. The linkage of health records is conducted in a systematic manner where all studies data are processed using the same data linkage, extraction and processing methods as a single 'pooled' sample; and the target data are collected, processed and cleaned to a standardised NHS protocol. This provides alignment of all linked EHRs across our selected studies. However, variations in the sample provided by the studies (e.g., exclusion criteria, study-specific patterns in participant permissions) may impact on the eventual linkage rates and that variations in the participants' personal identifiers used for linkage (e.g., the quality, completeness and temporal accuracy of the identifiers) may also introduce study-specific linkage rates. Together, these factors may impact on the 'pooling' of the linked data and may introduce bias.

The pooling of the longitudinal population studies' self-reported data was conducted in three parts. First, we undertook a scoping assessment of all variables available across all the potential longitudinal population studies to identify measures that were consistent. Some questions were not worded in exactly the same way, but worded close enough that they were comparable and consistent. At this point, any longitudinal population studies held in the UK LLC resource where we could not find consistent measures for our exposures and covariates were excluded.

Second, we undertook a process of harmonisation of variables so that their observations were directly comparable. This involved cleaning datasets and modifying the values so that they were consistent across all studies. A full description of the harmonisation process for our exposure variables (measures of healthcare disruption) is described in Appendix Table B. For the other covariates, the level of cleaning was mixed. Age, sex, and self-rated health were all directly comparable across all studies as they all used the same measure. Ethnicity was more challenging as different longitudinal population studies used a different range of categories. Some studies collected more detailed categories, whereas others had fewer categories. We opted to collapse ethnic groups into a single binary of 'White' or 'ethnic minority' because this was the only way of ensuring comparable groups. This was largely due to the inclusion of English Longitudinal Study of Ageing which did not collect any additional information. Housing tenure was also challenging due to similar issues where

studies had inconsistent categories for recording tenure (e.g., some studies did not split out renting into private or social). Similar to ethnicity, we defined categories that were consistent and allowed each study's values to be nested within so they could be comparable. Here we opted for whether the participant's household was either (i) owned outright or owned with mortgage, or (ii) other (i.e., rented or other arrangement). Health conditions were only selected where they were directly comparable, resulting in the selection of four conditions only. Other covariates used were gathered through the data linkage process meaning that they were consistent across studies. This process was necessary so that all of the longitudinal studies mapped onto each other meaning that their pooling was appropriate.

Third, we joined all the longitudinal population studies together into a single data object. This data object was linked to the pooled participant EHR records using an internal UK LLC participant ID number. We adjusted for their different sample frames (i.e., sample weights, primary sampling units, strata and finite population correction factor were adjusted for) in any analyses to account for their differing designs.

Table B: Description of healthcare disruption questions used to measure each exposure (after Maddock et al. 2021).

Cohort	Medication	Appointments	Procedures	Survey dates (response rates)
National Survey of Health and Development	Since the Coronavirus outbreak in March, have you had any difficulty obtaining any of your prescribed medication?	Q1: At the time of the Coronavirus outbreak in March, did you have an in-patient or out-patient appointment booked at a hospital for a consultation, investigation, treatment or surgery? Q2: Have you now had your in/ out-patient hospital appointment for a consultation, investigation or treatment? Q3: Did your (last) appointment take place on the planned date or was it delayed? Q4: Why has your in-/out-patient hospital appointment for a consultation, investigation or treatment not taken place?	Q1: At the time of the Coronavirus outbreak in March, did you have an in-patient or out-patient appointment booked at a hospital for a consultation, investigation, treatment or surgery? Q2: Have you now had your surgery? Q3: Did your (last) surgery take place on the planned date or was it delayed? Q4: Why has your surgery not taken place?	Three surveys: (i) May 2020 (68%), (ii) Sept to Oct 2020 (62%), and (iii) Jan to Feb 2021 (90%)
National Child Development Study				Three surveys: (i) May 2020 (60%), (ii) Sept to Oct 2020 (54%), and (iii) Jan to Feb 2021 (59%).
1970 British Cohort Study				Three surveys: (i) May 2020 (40%), (ii) Sept to Oct 2020 (44%), and (iii) Jan to Feb 2021 (45%).
Next Steps				Three surveys: (i) May 2020 (20%), (ii) Sept to Oct 2020 (32%), and (iii) Jan to Feb 2021 (34%).
Millennium Cohort Study				Three surveys: (i) May 2020 (27%), (ii) Sept to Oct 2020 (24%), and (iii) Jan to Feb 2021 (33%).
English Longitudinal Study of Ageing	Since the coronavirus outbreak, have you been able to get	Q1: Since the coronavirus outbreak, have you wanted to see or talk to a GP?	Since the coronavirus outbreak, have you had a hospital	Two surveys: (i) June to July 2020 (75%), and (ii) Nov to Dec 2020 (75%).

	access to your regular medications?	Q2: Have you been able to see or talk to a GP?	operation or treatment cancelled?	
Understanding Society	Q1: Still thinking about your situation now, have you been able to access the NHS services you need: Prescription medicine?<br>Q2: Still thinking about your situation now, have you been able to access the community health and social care services and support you need... Over the counter medications?	Thinking about your situation now, have you been able to access the NHS services you need to help manage your condition(s) over the last 4 weeks?<br>Q1: GP or primary care practice staff?<br>Q2: Hospital or clinic outpatient?<br>Q3: Hospital or clinic inpatient?<br>Q4: [since previous survey] have you had or been waiting for NHS treatment? Please select all that apply.	Q1: [since previous survey] have you had or been waiting for NHS treatment? Please select all that apply.<br>Q2: Has your treatment plan(s) been changed in any way?	Eight surveys. In 2020: (i) April (40%), (ii) May (34%), (iii) June (32%), (iv) July (31%), (v) Sept (29%), (vi) Nov (27%). In 2021: (vii) Jan (27%), (viii) Mar (29%).

Note on variable definitions based on Table C: Consistent variables across longitudinal population studies were defined following the approach used by Maddock et al. 2022. Variables were harmonising as follows (we refer to first five rows as the 'CLS cohorts' below since they had the same questionnaires run across each birth cohort): (i) *Disrupted access to medications*: reported disruption (value 1) was defined as participants recording 'yes' to any of the questions. A value of 0 (no disruption) was defined based on individuals recording 'no'. (ii) *Disrupted access to appointments*: For English Longitudinal Study of Ageing, a value of 1 (disruption experienced) was defined as people who responded that since the COVID-19 outbreak they had wanted to see or talk to a GP (Q1 value 1/yes) and that they were unable to see or talk to a GP (Q2 value 2/no). A value of 0 (no disruption) was recorded for other values. For Understanding Society, we recorded a value of 1 (disruption experienced) where people reported for Q1, Q2 or Q3 that they were unable to access staff, appointments were cancelled or an alternative was provided to seeing who they wanted (values 3-5), or for Q4 and Q5 that they recorded that consultations were cancelled or postponed

(values 1-3). A value of 0 (no disruption) was recorded for other values. For the CLS cohorts, a value of 1 (disruption experienced) was defined as where participants reported that they had an appointment booked (Q1 value 1/yes), that the appointment had happened (Q2 value 1/yes) and that the appointment was delayed (Q3 value 2) OR participants reported that they had an appointment booked (Q1 value 1/yes), that the appointment had not happened (Q2 value 2/no) and the reason the appointment had not happened was that it had been postponed or cancelled (Q4 values 1 or 3). A value of 0 (no disruption) was recorded for all other values. (iii) *Disrupted access to procedures*: For English Longitudinal Study of Ageing, a value of 1 (experienced disruption) was recorded for participants who responded with 'yes' and a value of 0 (no disruption) was record for people who responded with 'no'. For Understanding Society, a value of 1 (disruption experienced) was recorded for people who responded that they had planned treatment (Q1 values 2-4) and that this treatment was cancelled or postponed (Q2 values 1 or 3). A value of 0 (no disruption) was recorded for all other values. For the CLS cohorts, a value of 1 (disruption experienced) was defined as either people who had surgery booked (Q1 value 2), they had received the surgery (Q2 value 1) and that the surgery was delayed (Q3 value 2) OR they had surgery booked (Q1 value 2), the surgery had no taken placed (Q2 value 2) and the reason why it had not taken placed yet was that it had been postponed or cancelled (Q4 values 1 or 3). A value of 0 (no disruption) was defined otherwise. (iv) *Any disruption*: any experience of disrupted access to healthcare was defined based on a reported occurrence (value 1) across any of the measures in (i), (ii) or (iii). A value of 0 was given to all other cases. This was calculated after imputation of missing data in the other variables.

Table C: Missing data in pooled analytical sample across outcome, exposure and control variables.

Variable	Frequency	Percentage
Age	0	0.0
Sex	63	0.2
Ethnicity	994	3.4
Housing tenure	3349	11.4
Self-rated health	463	1.6
Index of multiple deprivation	193	0.7
Charlson Comorbidity Index (Quan weights)	0	0.0
Elixhauser Comorbidity Index	0	0.0
Asthma	15	0.1
Cancer	15	0.1
Diabetes	15	0.1
Hypertension	15	0.1
COVID-19	43	0.1
Disruption to appointments	1460	5.0
Disruption to medications	8919	30.5
Disruption to procedures	5513	18.8

Table D: Percentage of respondents who self-reported experiences of healthcare disruption between individuals who could and could not be linked to NHS Digital Hospital Episode Statistics.

Variable disruption to	Linked records (n)	Linked records (%)	Unlinked records (n)	Unlinked records (%)
Appointments	7124	25.4	1212	23.3
Medications	1040	5.0	167	4.6
Procedures	4324	18.0	684	15.4

Table E: Model summary statistics for a logistic (binomial) regression exploring associations between experiences of healthcare disruption and whether an individual had an avoidable hospitalisation.

Model	Odds Ratio	Lower 95% CI	Higher 95% CI	P value
Any ambulatory care sensitive				
Unadjusted	2.97	1.92	4.57	<0.001
Adjusted	1.80	1.39	2.34	<0.001
Acute ambulatory care sensitive				
Unadjusted	2.25	1.57	3.25	<0.001
Adjusted	2.01	1.39	2.92	<0.001
Chronic ambulatory care sensitive				
Unadjusted	3.10	2.25	4.26	<0.001
Adjusted	1.80	1.31	2.48	<0.001
Vaccine-preventable ambulatory care sensitive				
Unadjusted	4.06	0.87	18.92	0.075

Adjusted	1.35	0.70	2.59	0.368
Emergency urgent care sensitive				
Unadjusted	1.80	1.38	2.36	<0.001
Adjusted	1.17	0.88	1.57	0.269
Any hospital admission				
Unadjusted	2.80	2.46	3.22	<0.001
Adjusted	1.82	1.55	2.14	<0.001

Table F: Model summary statistics for a logistic (binomial) regression exploring associations between experiences of three types of healthcare disruption (procedures, medications and appointments) and whether an individual had an avoidable hospitalisation (by type).

Model	Odds Ratio	Lower 95% CI	Higher 95% CI	P value
Any ambulatory care sensitive - unadjusted model				
Appointments	1.75	1.25	2.48	0.001
Medications	2.80	0.84	9.39	0.096
Procedures	2.53	1.70	3.78	<0.001
Any ambulatory care sensitive - adjusted model				
Appointments	1.52	1.09	2.12	0.013
Medications	2.29	1.02	5.10	0.044
Procedures	1.77	1.30	2.41	<0.001
Acute ambulatory care sensitive - unadjusted model				
Appointments	1.46	0.86	2.48	0.163
Medications	1.09	0.39	3.06	0.864
Procedures	1.92	1.04	3.53	0.038
Acute ambulatory care sensitive - adjusted model				
Appointments	1.38	0.84	2.23	0.203
Medications	1.07	0.36	3.25	0.898
Procedures	1.77	0.97	3.22	0.064
Chronic ambulatory care sensitive - unadjusted model				
Appointments	1.82	1.17	2.80	0.007
Medications	0.62	0.33	1.17	0.144
Procedures	2.80	1.79	4.39	<0.001
Chronic ambulatory care sensitive - adjusted model				
Appointments	1.43	0.95	2.16	0.085
Medications	0.59	0.26	1.35	0.209
Procedures	1.88	1.28	2.75	0.001
Vaccine-preventable ambulatory care sensitive - unadjusted model				
Appointments	2.20	0.99	4.90	0.054
Medications				
Procedures	3.32	1.22	8.94	0.018
Vaccine-preventable ambulatory care sensitive - adjusted model				
Appointments	1.79	0.83	3.86	0.138

Medications				
Procedures	1.40	0.82	2.41	0.213
Emergency urgent care sensitive - unadjusted model				
Appointments	1.23	0.90	1.72	0.201
Medications	0.84	0.52	1.35	0.47
Procedures	2.01	1.42	2.89	<0.001
Emergency urgent care sensitive - adjusted model				
Appointments	1.01	0.75	1.38	0.935
Medications	0.82	0.45	1.48	0.502
Procedures	1.45	1.05	1.99	0.022
Any hospital admission - unadjusted model				
Appointments	1.72	1.43	2.05	<0.001
Medications	0.90	0.58	1.39	0.63
Procedures	2.23	1.84	2.69	<0.001
Any hospital admission - adjusted model				
Appointments	1.46	1.21	1.75	<0.001
Medications	0.86	0.49	1.51	0.594
Procedures	1.57	1.28	1.92	<0.001

Table G: Summary of outcome variables across two sensitivity analyses.

**Measure**	**1: Include events post survey (events)**			**2: Survival analysis (days)**	
	**Frequency**	**Percent**	**Weighted Percent**	**Unweighted Mean (std dev)**	**Weighted Mean (std dev)**
Total admissions	2462	8.41	9.30	205 (141)	199 (139)
Ambulatory care sensitive any	503	1.72	2.44	217 (136)	215 (125)
Ambulatory care sensitive acute	218	0.74	0.90	219 (132)	224 (131)
Ambulatory care sensitive chronic	240	0.82	0.95	212 (139)	193 (138)
Ambulatory care sensitive vaccine preventable	60	0.20	0.67	239 (142)	241 (85)
Emergency urgent care sensitive	371	1.27	1.42	220 (140)	216 (139)

Note: std dev = standard deviation

Table H: Logistic regression results for a sensitivity analysis excluding outcome events that occurred before the last known survey date to examine the association between experiences of healthcare disruption to avoidable hospitalisations (i.e., results presented in Table 3).

**Model**	**Odds Ratio**	**Lower 95% CI**	**Higher 95% CI**	**P value**
Any ambulatory care sensitive				

Unadjusted	2.96	1.65	5.30	<0.001
Adjusted	1.68	1.22	2.31	0.002
Acute ambulatory care sensitive				
Unadjusted	1.97	1.21	3.21	0.007
Adjusted	1.72	1.05	2.80	0.03
Chronic ambulatory care sensitive				
Unadjusted	3.06	2.05	4.55	<0.001
Adjusted	1.80	1.22	2.65	0.003
Vaccine-preventable ambulatory care sensitive				
Unadjusted	4.71	0.84	26.47	0.078
Adjusted	1.34	0.67	2.66	0.412
Emergency urgent care sensitive				
Unadjusted	1.78	1.26	2.51	0.001
Adjusted	1.19	0.84	1.69	0.33
Any hospital admission				
Unadjusted	2.50	2.12	2.95	<0.001
Adjusted	1.62	1.34	1.96	<0.001

Table I: Cox regression results for a sensitivity analysis for time to hospital admission (month) of events happening after the last known survey date to examine the association between experiences of healthcare disruption to avoidable hospitalisations (i.e., results presented in Table 3).

Model	Hazards Ratio	Lower 95% CI	Higher 95% CI	P value
Any ambulatory care sensitive				
Unadjusted	2.14	1.80	2.55	<0.001
Adjusted	1.44	1.19	1.73	<0.001
Acute ambulatory care sensitive				
Unadjusted	1.84	1.41	2.41	<0.001
Adjusted	1.59	1.19	2.10	0.001
Chronic ambulatory care sensitive				
Unadjusted	2.48	1.92	3.20	<0.001
Adjusted	1.45	1.11	1.89	0.007
Vaccine-preventable ambulatory care sensitive				
Unadjusted	2.05	1.24	3.41	0.005
Adjusted	0.96	0.56	1.62	0.867
Emergency urgent care sensitive				
Unadjusted	1.92	1.57	2.36	<0.001
Adjusted	1.32	1.06	1.63	0.012
Any hospital admission				
Unadjusted	2.57	2.37	2.78	<0.001
Adjusted	1.72	1.58	1.87	<0.001

Table J: Logistic regression results for a sensitivity analysis excluding outcome events that occurred before the last known survey date to examine the association between experiences of healthcare disruption to avoidable hospitalisations (i.e., results presented in Table 4).

Model	Odds Ratio	Lower 95% CI	Higher 95% CI	P value
Any ambulatory care sensitive - unadjusted model				
Appointments	1.62	1.03	2.56	0.038
Medications	3.82	1.05	14.01	0.042
Procedures	2.53	1.49	4.31	0.001
Any ambulatory care sensitive - adjusted model				
Appointments	1.39	0.91	2.12	0.12
Medications	3.03	1.32	6.96	0.009
Procedures	1.67	1.12	2.46	0.012
Acute ambulatory care sensitive - unadjusted model				
Appointments	1.03	0.51	2.12	0.932
Medications	1.23	0.32	4.76	0.764
Procedures	1.99	0.84	4.66	0.116
Acute ambulatory care sensitive - adjusted model				
Appointments	1.00	0.55	1.80	0.99
Medications	1.28	0.30	5.47	0.738
Procedures	1.77	0.79	3.90	0.162
Chronic ambulatory care sensitive - unadjusted model				
Appointments	1.86	1.02	3.39	0.042
Medications	0.73	0.34	1.54	0.409
Procedures	2.48	1.32	4.66	0.005
Chronic ambulatory care sensitive - adjusted model				
Appointments	1.45	0.84	2.53	0.189
Medications	0.69	0.27	1.79	0.445
Procedures	1.73	1.04	2.86	0.034
Vaccine-preventable ambulatory care sensitive - unadjusted model				
Appointments	2.34	0.93	5.87	0.071
Medications				
Procedures	3.60	1.17	11.02	0.025
Vaccine-preventable ambulatory care sensitive - adjusted model				
Appointments	1.75	0.75	4.14	0.199
Medications				
Procedures	1.35	0.70	2.59	0.362
Emergency urgent care sensitive - unadjusted model				
Appointments	1.19	0.74	1.92	0.468
Medications	0.76	0.42	1.39	0.381
Procedures	1.95	1.14	3.32	0.014
Emergency urgent care sensitive - adjusted model				
Appointments	1.03	0.68	1.57	0.888

Medications	0.75	0.37	1.54	0.433
Procedures	1.38	0.90	2.12	0.147
Any hospital admission - unadjusted model				
Appointments	1.48	1.20	1.82	<0.001
Medications	1.05	0.66	1.68	0.834
Procedures	2.18	1.75	2.72	<0.001
Any hospital admission - adjusted model				
Appointments	1.28	1.04	1.58	0.021
Medications	1.04	0.59	1.84	0.888
Procedures	1.54	1.22	1.93	<0.001

Note: Results for vaccine-preventable ambulatory care sensitive conditions and disruption to medications were not robust due to small number issues.

Table K: Cox regression results for a sensitivity analysis excluding outcome events that occurred before the last known survey date to examine the association between experiences of healthcare disruption to avoidable hospitalisations (i.e., results presented in Table 4).

Model	Hazards Ratio	Lower 95% CI	Higher 95% CI	P value
Any ambulatory care sensitive - unadjusted model				
Appointments	1.51	1.38	1.67	<0.001
Medications	0.96	0.82	1.14	0.669
Procedures	2.25	2.03	2.48	<0.001
Any ambulatory care sensitive - adjusted model				
Appointments	1.31	1.20	1.43	<0.001
Medications	1.21	1.02	1.43	0.026
Procedures	1.68	1.52	1.84	<0.001
Acute ambulatory care sensitive - unadjusted model				
Appointments	1.51	1.36	1.67	<0.001
Medications	0.96	0.82	1.14	0.66
Procedures	2.25	2.03	2.48	<0.001
Acute ambulatory care sensitive - adjusted model				
Appointments	1.31	1.20	1.45	<0.001
Medications	1.20	1.01	1.42	0.035
Procedures	1.67	1.52	1.84	<0.001
Chronic ambulatory care sensitive - unadjusted model				
Appointments	1.51	1.36	1.67	<0.001
Medications	0.96	0.82	1.14	0.65
Procedures	2.25	2.03	2.48	<0.001
Chronic ambulatory care sensitive - adjusted model				
Appointments	1.31	1.20	1.43	<0.001
Medications	1.20	1.02	1.42	0.032
Procedures	1.68	1.52	1.84	<0.001
Vaccine-preventable ambulatory care sensitive - unadjusted model				
Appointments	1.51	1.36	1.67	<0.001

Medications				
Procedures	2.25	2.03	2.48	<0.001
Vaccine-preventable ambulatory care sensitive - adjusted model				
Appointments	1.31	1.20	1.43	<0.001
Medications				
Procedures	1.68	1.52	1.84	<0.001
Emergency urgent care sensitive - unadjusted model				
Appointments	1.51	1.36	1.67	<0.001
Medications	0.97	0.82	1.14	0.679
Procedures	2.25	2.05	2.48	<0.001
Emergency urgent care sensitive - adjusted model				
Appointments	1.31	1.20	1.43	<0.001
Medications	1.21	1.02	1.42	0.029
Procedures	1.68	1.52	1.84	<0.001
Any hospital admission - unadjusted model				
Appointments	1.51	1.36	1.67	<0.001
Medications	0.97	0.82	1.14	0.688
Procedures	2.27	2.05	2.51	<0.001
Any hospital admission - adjusted model				
Appointments	1.31	1.20	1.43	<0.001
Medications	1.20	1.01	1.42	0.035
Procedures	1.68	1.52	1.84	<0.001

Note: Results for vaccine-preventable ambulatory care sensitive conditions and disruption to medications were not robust due to small number issues.

Table L: Logistic regression results for a sensitivity analysis replacing Charlson Comorbidity Index with Elixhauser Index as a control variable when analysing the association between any healthcare disruption and avoidable hospitalisations (i.e., results presented in Table 3).

Model	Odds Ratio	Lower 95% CI	Higher 95% CI	P value
Any ambulatory care sensitive	1.70	1.28	2.26	<0.001
Acute ambulatory care sensitive	1.92	1.30	2.83	0.001
Chronic ambulatory care sensitive	1.58	1.13	2.21	0.007
Vaccine-preventable ambulatory care sensitive	1.33	0.71	2.50	0.377
Emergency urgent care sensitive	1.10	0.82	1.48	0.511
Any hospital admission	1.69	1.45	1.98	<0.001

Table M: Logistic regression results for a sensitivity analysis replacing Charlson Comorbidity Index with Elixhauser Index as a control variable when analysing the association between type of healthcare disruption and avoidable hospitalisations (i.e., results presented in Table 4).

Model	Odds Ratio	Lower 95% CI	Higher 95% CI	P value

Any ambulatory care sensitive				
Appointments	1.54	1.07	2.18	0.019
Medications	2.32	1.00	5.42	0.051
Procedures	1.63	1.17	2.27	0.004
Acute ambulatory care sensitive				
Appointments	1.35	0.83	2.20	0.229
Medications	1.08	0.36	3.22	0.884
Procedures	1.68	0.89	3.16	0.11
Chronic ambulatory care sensitive				
Appointments	1.42	0.92	2.16	0.11
Medications	0.62	0.28	1.35	0.227
Procedures	1.58	1.08	2.34	0.019
Vaccine-preventable ambulatory care sensitive				
Appointments	1.68	0.85	3.32	0.135
Medications				
Procedures	1.45	0.83	2.53	0.192
Emergency urgent care sensitive				
Appointments	1.00	0.73	1.36	0.988
Medications	0.80	0.44	1.46	0.476
Procedures	1.36	0.99	1.88	0.06
Any hospital admission				
Appointments	1.43	1.19	1.75	<0.001
Medications	0.84	0.48	1.48	0.544
Procedures	1.45	1.17	1.77	<0.001

Table N: Logistic regression results for a sensitivity analysis further controlling for presence of asthma, cancer, diabetes and hypertension when analysing the association between any healthcare disruption and avoidable hospitalisations (i.e., results presented in Table 3).

Model	Odds Ratio	Lower 95% CI	Higher 95% CI	P value
Any ambulatory care sensitive	1.69	1.32	2.17	<0.001
Acute ambulatory care sensitive	1.96	1.35	2.85	<0.001
Chronic ambulatory care sensitive	1.69	1.23	2.34	0.001
Vaccine-preventable ambulatory care sensitive	1.12	0.65	1.93	0.688
Emergency urgent care sensitive	1.15	0.87	1.53	0.338
Any hospital admission	1.72	1.47	2.02	<0.001

Table O: Logistic regression results for a sensitivity analysis further controlling for presence of asthma, cancer, diabetes and hypertension when analysing the association between type of healthcare disruption and avoidable hospitalisations (i.e., results presented in Table 4).

Model	Odds Ratio	Lower 95% CI	Higher 95% CI	P value

Any ambulatory care sensitive				
Appointments	1.36	1.02	1.84	0.039
Medications	2.05	1.04	4.06	0.039
Procedures	1.72	1.27	2.34	0.001
Acute ambulatory care sensitive				
Appointments	1.31	0.79	2.16	0.299
Medications	0.97	0.30	3.16	0.954
Procedures	1.73	0.94	3.19	0.075
Chronic ambulatory care sensitive				
Appointments	1.36	0.90	2.05	0.138
Medications	0.54	0.22	1.35	0.189
Procedures	1.80	1.23	2.64	0.002
Vaccine-preventable ambulatory care sensitive				
Appointments	1.27	0.69	2.34	0.445
Medications				
Procedures	1.34	0.82	2.18	0.248
Emergency urgent care sensitive				
Appointments	0.98	0.73	1.34	0.91
Medications	0.79	0.41	1.48	0.457
Procedures	1.43	1.04	1.97	0.025
Any hospital admission				
Appointments	1.39	1.15	1.68	0.001
Medications	0.81	0.43	1.52	0.508
Procedures	1.51	1.22	1.84	<0.001

Table P: Logistic regression model results for a falsification test predicting whether an individual had been fully vaccinated for COVID-19 (two doses) or not.

Model	Odds Ratio	Lower 95% CI	Higher 95% CI	P value
Any healthcare disruption				
Unadjusted	1.47	1.11	1.94	0.007
Adjusted	1.05	0.84	1.32	0.673
Healthcare disruption type - unadjusted				
Appointments	1.52	1.03	2.25	0.035
Medications	0.48	0.26	0.89	0.019
Procedures	1.33	0.81	2.19	0.255
Healthcare disruption type - adjusted				
Appointments	1.35	0.79	2.29	0.271
Medications	0.63	0.35	1.13	0.122
Procedures	1.00	0.58	1.72	0.991

Note: Estimates are Odds Ratios. CI = Confidence Interval. Model adjustment includes the following variables: age, age-squared, sex, ethnicity, housing tenure, self-rated health status, Charlson Comorbidity Index and longitudinal cohort).

Table Q: Logistic regression results for a complete case analysis on the association between any healthcare disruption and avoidable hospitalisations (i.e., results presented in Table 3).

Model	Odds Ratio	Lower 95% CI	Higher 95% CI	P value
Any ambulatory care sensitive				
Unadjusted	2.83	1.75	4.57	<0.001
Adjusted	1.82	1.25	2.66	0.002
Acute ambulatory care sensitive				
Unadjusted	1.90	1.30	2.80	0.001
Adjusted	1.67	1.05	2.64	0.03
Chronic ambulatory care sensitive				
Unadjusted	3.06	2.16	4.35	<0.001
Adjusted	1.48	0.93	2.36	0.096
Vaccine-preventable ambulatory care sensitive				
Unadjusted	4.53	0.96	21.54	0.057
Adjusted	2.44	1.15	5.21	0.02
Emergency urgent care sensitive				
Unadjusted	1.60	1.23	2.08	<0.001
Adjusted	1.11	0.80	1.52	0.552
Any hospital admission				
Unadjusted	2.86	2.48	3.29	<0.001
Adjusted	1.97	1.65	2.36	<0.001

Table R: Logistic regression results for a complete case analysis on the association between type of healthcare disruption and avoidable hospitalisations (i.e., results presented in Table 4).

Model	Odds Ratio	Lower 95% CI	Higher 95% CI	P value
Any ambulatory care sensitive - unadjusted model				
Appointments	1.28	0.99	1.67	0.059
Medications	1.01	0.63	1.62	0.957
Procedures	1.97	1.52	2.56	<0.001
Any ambulatory care sensitive - adjusted model				
Appointments	1.20	0.91	1.57	0.192
Medications	0.93	0.54	1.62	0.809
Procedures	1.52	1.17	1.97	0.002
Acute ambulatory care sensitive - unadjusted model				
Appointments	1.58	1.06	2.36	0.024
Medications	1.03	0.46	2.32	0.947
Procedures	1.70	1.13	2.56	0.012
Acute ambulatory care sensitive - adjusted model				
Appointments	1.40	0.90	2.18	0.131
Medications	0.59	0.23	1.49	0.264

Procedures	1.58	1.03	2.44	0.037
Chronic ambulatory care sensitive - unadjusted model				
Appointments	1.27	0.90	1.80	0.174
Medications	1.07	0.59	1.95	0.809
Procedures	2.51	1.77	3.60	<0.001
Chronic ambulatory care sensitive - adjusted model				
Appointments	1.16	0.82	1.67	0.4
Medications	1.34	0.66	2.69	0.417
Procedures	1.70	1.21	2.39	0.002
Vaccine-preventable ambulatory care sensitive - unadjusted model				
Appointments	0.68	0.34	1.34	0.256
Medications				
Procedures	1.54	0.79	2.97	0.2
Vaccine-preventable ambulatory care sensitive - adjusted model				
Appointments	0.71	0.37	1.36	0.309
Medications				
Procedures	1.09	0.59	2.03	0.771
Emergency urgent care sensitive - unadjusted model				
Appointments	1.04	0.78	1.39	0.784
Medications	1.32	0.84	2.10	0.227
Procedures	2.05	1.52	2.75	<0.001
Emergency urgent care sensitive - adjusted model				
Appointments	0.96	0.70	1.31	0.789
Medications	1.27	0.74	2.18	0.381
Procedures	1.65	1.23	2.20	0.001
Any hospital admission - unadjusted model				
Appointments	1.32	1.09	1.60	0.004
Medications	1.31	0.88	1.93	0.192
Procedures	2.66	2.20	3.22	<0.001
Any hospital admission - adjusted model				
Appointments	1.12	0.92	1.35	0.24
Medications	1.31	0.87	1.99	0.199
Procedures	2.34	1.93	2.80	<0.001

Table S: Detailed information of the seven longitudinal population studies.

BCS70: 1970 British Cohort Study	
**Description of Study Population** (including citations and references if required)	The 1970 British Cohort Study (BCS70) follows the lives of more than 17,000 people born in England, Scotland and Wales in a single week of 1970. Over the course of cohort members’ lives, BCS70 has collected information on health, physical, educational and social development, and economic circumstances, among other factors.

	Since the birth survey in 1970, there have been nine 'sweeps' of all cohort members at ages 5, 10, 16, 26, 30, 34, 38, 42 and most recently at 46 (a biomedical data collection). The Age 51 Sweep is currently in the field (2022).<br><br>Data have been collected from a number of different sources, including the midwife present at birth, parents of the cohort members, head and class teachers, school health service personnel and the cohort members themselves.<br><br>The data have been collected in a variety of ways, including via paper and electronic questionnaires, clinical records, medical examinations, biological samples, physical measurements, tests of ability, educational assessments and diaries.<br><br>The study is conducted by the Centre for Longitudinal Studies.
**Acknowledgements**	BCS70 is core-funded by the ESRC.
**Ethics**	Ethics approval has been obtained for each follow-up from an NHS Research Ethics Committee (REC) since 2000. In addition, separate REC approval is in place to cover the ongoing activities of the study in between major sweeps of data collection (i.e. Keeping in touch with and tracing cohort members; cleaning, documenting and providing access to the data for research; and linking data from administrative sources to survey data to increase the utility of the data for research).
**Website for Data Requests**	https://cls.ucl.ac.uk/cls-studies/bcs70/
**COVID-19 survey dates**	Three surveys: May 2020, Sept-Oct 2020 and Feb-Mar 2021
**Citation**	University College London, UCL Institute of Education, Centre for Longitudinal Studies. (2022). COVID-19 Survey in Five National Longitudinal Cohort Studies: Millennium Cohort Study, Next Steps, 1970 British Cohort Study and 1958 National Child Development Study, 2020-2021. [data collection]. 4th Edition. UK Data Service. SN: 8658, DOI: 10.5255/UKDA-SN-8658-4

**ELSA: English Longitudinal Study of Ageing**

**Description of Study Population** (including citations and references if required)	The English Longitudinal Study of Ageing (ELSA) is a unique and rich resource of information on the dynamics of health, social, wellbeing and economic circumstances in the English population aged 50 and older [1].<br><br>The original sample was drawn from households that had previously responded to the Health Survey for England (HSE) between 1998 and 2001. The main fieldwork began in March 2002. The same group of respondents have been interviewed at two-yearly interviews .<br><br>[1]Banks J, Batty GD, Breedvelt JJF, Coughlin K, Crawford R, Marmot M, Nazroo J, Oldfield Z, Steel N, Steptoe A, Wood M, Zaninotto P (2021) English Longitudinal Study of Ageing: Waves 0-9, 1998-2019
**Acknowledgements**	The English Longitudinal Study of Ageing was developed by a team of researchers based at University College London, NatCen Social Research, the Institute for Fiscal Studies, the University of Manchester and the University of East Anglia. The data were collected by NatCen Social Research. The funding is currently provided by the National Institute on Aging (Ref: R01AG017644) and by a consortium of UK government departments: Department for Health and Social Care; Department for Transport; Department for Work and Pensions, which is coordinated by the National Institute for Health Research (NIHR, Ref: 198-1074). Funding has also been provided by the Economic and Social Research Council (ESRC).
**Ethics**	https://www.elsa-project.ac.uk/ethical-approval
**Website for Data Requests**	https://www.elsa-project.ac.uk/data-and-documentation
**COVID-19 survey dates**	Two surveys: Jun-Jul 2020 and Nov-Dec 2020
**Citation**	Marmot, M., Pacchiotti, B., Banks, J., Steel, N., Oldfield, Z., Nazroo, J., Dangerfield, P., Coughlin, K., Zaninotto, P., Crawford, R., Steptoe, A., Addario, G., Wood, M., Batty, G. David. (2022). English Longitudinal Study of Ageing COVID-19 Study, Waves 1-2, 2020. [data collection]. 3rd Edition. UK Data Service. SN: 8688, DOI: 10.5255/UKDA-SN-8688-3

**MCS: Millennium Cohort Study**

**Description of Study Population** (including citations and references if required)	The Millennium Cohort Study (MCS) is following the lives of young people born across England, Scotland, Wales and Northern Ireland in 2000-02. The study began with an original sample of 18,818 cohort members. The study is designed and led by the Centre for Longitudinal Studies (CLS) at University College London.<br><br>The broad aim of the study is to examine the impact that circumstances and experiences at one stage of life have on outcomes and achievements in later life. Since the baseline survey at age 9 months, there have been six major 'sweeps' at ages 3, 5, 7, 11, 14 and 17. The next sweep, at age 22, is currently under development.<br><br>Data have been collected from a number of different sources, including the cohort members and their parents and teachers. The data have been collected in a variety of ways, including via paper and electronic questionnaires, biological samples, physical measurements, tests of ability, and linked educational attainment and health records.<br><br>The information collected forms a high quality data resource for scientific investigations across a full range of domains of individuals' lives and across different points in time in them. The study has been designed to ensure comparability with other major cohort studies both in the UK and internationally and to permit the examination of links between social change and the changing experiences of different cohorts.<br><br>https://www.llcsjournal.org/index.php/llcs/article/view/410/0<br><br>https://academic.oup.com/ije/article/43/6/1719/703283
**Acknowledgements**	MCS is core-funded by the ESRC and co-funded by a consortium of government departments.
**Ethics**	Ethics approval has been obtained for each follow-up from an NHS Research Ethics Committee (REC). In addition, separate REC approval is in place to cover the ongoing activities of the study in between major sweeps of data collection (i.e. Keeping in touch with and tracing cohort Members; cleaning, documenting and providing access to the data for research and linking data from administrative sources to survey data to increase the utility of the data for research.
**Website for Data Requests**	https://cls.ucl.ac.uk/cls-studies/mcs/

**COVID-19 survey dates**	Three surveys: May 2020, Sept-Oct 2020 and Feb-Mar 2021
**Citation**	University College London, UCL Institute of Education, Centre for Longitudinal Studies. (2022). COVID-19 Survey in Five National Longitudinal Cohort Studies: Millennium Cohort Study, Next Steps, 1970 British Cohort Study and 1958 National Child Development Study, 2020-2021. [data collection]. 4th Edition. UK Data Service. SN: 8658, DOI: 10.5255/UKDA-SN-8658-4

**NCDS: National Child Development Study**	
**Description of Study Population** (including citations and references if required)	The National Child Development Study (NCDS) is a continuing longitudinal study that seeks to follow the lives of all those living in Great Britain who were born in one particular week in 1958. Conducted by the Centre for Longitudinal Studies , the aim of the study is to improve understanding of the factors affecting human development over the whole lifespan. It collects information on physical and educational development, economic circumstances, employment, family life, health behaviour, wellbeing, social participation and attitudes.<br><br>The broad aim of the study is to examine the impact that circumstances and experiences at one stage of life have on outcomes and achievements in later life. Since the birth survey in 1958, there have been ten ‘sweeps’ of all cohort members at ages 7, 11, 16, 23, 33, 42, 44/5 (a biomedical collection) 46, 50 and most recently at 55. The Age 62 Sweep is currently in the field (2022).<br><br>Data have been collected from a number of different sources, including the midwife present at birth, parents of the cohort members, teachers, doctors and the cohort members themselves. The data have been collected in a variety of ways, including via paper and electronic questionnaires, clinical records, medical examinations, biological samples, physical measurements, tests of ability and educational assessments.<br><br>The information collected forms a high quality data resource for scientific investigations across a full range of domains of individuals’ lives and across different points in time in them. The study has been designed to ensure comparability with other major cohort studies and to permit the examination of links between social change and the changing experiences of different cohorts.

	https://cls.ucl.ac.uk/cls-studies/1958-national-child-development-study/
**Acknowledgements**	NCDS is core-funded by the ESRC.
**Ethics**	Ethics approval has been obtained for each follow-up from an NHS Research Ethics Committee (REC) since 2000. In addition, separate REC approval is in place to cover the ongoing activities of the study in between major sweeps of data collection (i.e. Keeping in touch with and tracing cohort members; cleaning, documenting and providing access to the data for research; and linking data from administrative sources to survey data to increase the utility of the data for research).
**Website for Data Requests**	https://cls.ucl.ac.uk/cls-studies/ncds/
**COVID-19 survey dates**	Three surveys: May 2020, Sept-Oct 2020 and Feb-Mar 2021
**Citation**	University College London, UCL Institute of Education, Centre for Longitudinal Studies. (2022). COVID-19 Survey in Five National Longitudinal Cohort Studies: Millennium Cohort Study, Next Steps, 1970 British Cohort Study and 1958 National Child Development Study, 2020-2021. [data collection]. 4th Edition. UK Data Service. SN: 8658, DOI: 10.5255/UKDA-SN-8658-4

**Next Steps**	
**Description of Study Population** (including citations and references if required)	Next Steps (previously known as the Longitudinal Study of Young People in England (LSYPE1)) is a major longitudinal study that follows the lives of around 16,000 people born in 1989-90. The first seven sweeps of the study (2004-2010) were funded and managed by the Department for Education and mainly focused on the educational and early labour market experiences of young people.<br><br>The study began in 2004 and included young people in Year 9 who attended state and independent schools in England. Following the initial survey at age 13-14, the cohort members were interviewed every year until 2010.<br><br>In 2013 the management of Next Steps was transferred to the Centre for Longitudinal Studies (CLS) at the IOE, UCL's Faculty of Education and Society. The first sweep conducted by CLS aimed to find out how the lives of the cohort members had turned out at age 25. It maintained the strong focus on education, but the content was

	broadened to become a more multi-disciplinary research resource.<br><br>The Age 32 Sweep is currently in the field (2022).<br><br>https://doc.ukdataservice.ac.uk/doc/5545/mrdoc/pdf/next_steps_userguide_to_the_redeposit_of_sweeps_1to7_may2020.pdf<br><br>https://doc.ukdataservice.ac.uk/doc/5545/mrdoc/pdf/nextsteps_age25_survey_user_guide_v3.pdf<br><br>https://cls.ucl.ac.uk/cls-studies/next-steps/
**Acknowledgements**	Next Steps now is core-funded by the ESRC.
**Ethics**	Ethics approval is obtained for each follow-up from an NHS Research Ethics Committee (REC). In addition, separate REC approval is in place to cover the ongoing activities of the study in between major sweeps of data collection (i.e. keeping in touch with and tracing cohort members; cleaning, documenting and providing access to the data for research; and linking data from administrative sources to survey data to increase the utility of the data for research).
**Website for Data Requests**	https://cls.ucl.ac.uk/cls-studies/next-steps/
**COVID-19 survey dates**	Three surveys: May 2020, Sept-Oct 2020 and Feb-Mar 2021
**Citation**	University College London, UCL Institute of Education, Centre for Longitudinal Studies. (2022). COVID-19 Survey in Five National Longitudinal Cohort Studies: Millennium Cohort Study, Next Steps, 1970 British Cohort Study and 1958 National Child Development Study, 2020-2021. [data collection]. 4th Edition. UK Data Service. SN: 8658, DOI: 10.5255/UKDA-SN-8658-4

**NSHD: Medical Research Council National Survey of Health and Development**	
**Description of Study Population (including citations and references if required)**	The MRC National Survey of Health and Development (NSHD) is a socially stratified birth cohort of 2,547 women and 2,815 men. It is a sample of all births in England, Scotland, and Wales that occurred in one week in March 1946, and consists of all single births to married women with a husband in non-manual and agricultural employment and 1 in 4 of all comparable births to women with a husband in manual employment.[1]

	[1]Kuh et al. Cohort profile: updating the cohort profile for the MRC National Survey of Health and Development: a new clinic-based data collection for ageing research. Int J Epidemiol. 2011 Feb;40(1):e1-9. doi: 10.1093/ije/dyq231.
**Acknowledgements**	The UK Medical Research Council provides core funding for the MRC National Survey of Health and Development (MC_UU_00019/1). We are extremely grateful to the NSHD study members for their lifelong participation and continuing support; and to past and present members of the study teams, who helped to collect and process the data.
**Ethics**	Ethical approval for the study was obtained from the UK Research Ethics Committee (REC).
**Website for Data Requests**	https://skylark.ucl.ac.uk/
**COVID-19 survey dates**	Three surveys: May 2020, Sept-Oct 2020 and Feb-Mar 2021
**Citation**	University College London, MRC Unit for Lifelong Health and Ageing. (2021). COVID-19 Survey in Five National Longitudinal Cohort Studies: MRC National Survey of Health and Development, 2020-2021: Special Licence Access. [data collection]. 3rd Edition. UK Data Service. SN: 8732, DOI: 10.5255/UKDA-SN-8732-3

**Understanding Society – the UK Household Longitudinal Study**	
**Description of Study Population** (including citations and references if required)	Understanding Society, the UK Household Longitudinal Study, is a longitudinal survey of the members of ~40,000 households (at Wave 1, 2009-10) in the United Kingdom. The survey sample consists of a large General Population Sample (~26,000 households) plus three other components: the Ethnic Minority Boost Sample (~4,000 households), the former British Household Panel Survey sample (~8,000 households) and the Immigrant and Ethnic Minority Boost Sample (~2,900 households, added at Wave 6). Household and individual interviews are conducted annually. The study is multi-topic and multi-purpose.<br><br>From April 2020 to September 2021, participants from the main Understanding Society sample were asked to complete nine short web-surveys (with a telephone option in some months). The COVID-19 study covered the changing impact of the pandemic on the welfare of UK individuals, families and wider communities. ~18,000

	individuals provided a full or partial interview at Wave 1 (April 2020).<br><br>At Wave 8 of the COVID-19 study, 8477 participants provided consent to link their survey data to administrative health records.
**Acknowledgements**	Understanding Society is an initiative funded by the Economic and Social Research Council and various Government Departments, with scientific leadership by the Institute for Social and Economic Research, University of Essex, and survey delivery by NatCen Social Research and Kantar Public<br><br>The COVID-19 study (2020-2021) was funded by the Economic and Social Research Council and the Health Foundation. Serology testing was funded by the COVID-19 Longitudinal Health and Wealth – National Core Study. Fieldwork for the web survey was carried out by Ipsos MORI and for the telephone survey by Kantar.
**Ethics**	The University of Essex Ethics Committee has approved all data collection on Understanding Society main study, COVID-19 surveys and innovation panel waves, including asking consent for all data linkages except to health records.<br><br>Approval for asking consent for health record linkage and for the collection of blood and subsequent serology testing in the March 2021 wave of the COVID-19 study was obtained from London – City & East Research Ethics Committee (21/HRA/0644).
**Website for Data Requests**	https://ukllc.ac.uk or https://ukdataservice.ac.uk
**COVID-19 survey dates**	Eight surveys: Apr 2020, May 2020, Jun 2020, Jul 2020, Sept 2020, Nov 2020, Jan 2021, Mar 2021
**Citation**	University of Essex, Institute for Social and Economic Research. (2022). Understanding Society: Waves 1-12, 2009-2021 and Harmonised BHPS: Waves 1-18, 1991-2009. [data collection]. 17th Edition. UK Data Service. SN: 6614, http://doi.org/10.5255/UKDA-SN-6614-18.<br><br>University of Essex, Institute for Social and Economic Research. (2021). Understanding Society: COVID-19 Study, 2020-2021. [data collection]. 11th Edition. UK Data Service. SN: 8644, DOI: 10.5255/UKDA-SN-8644-11
